# Immunologic Roles of Hyaluronan in Dermal Wound Healing

**DOI:** 10.3390/biom11081234

**Published:** 2021-08-18

**Authors:** Aditya Kaul, Walker D. Short, Sundeep G. Keswani, Xinyi Wang

**Affiliations:** Laboratory for Regenerative Tissue Repair, Division of Pediatric Surgery, Department of Surgery, Texas Children’s Hospital/Baylor College of Medicine, Houston, TX 77030, USA; adit.kaul@gmail.com (A.K.); wdshort@bcm.edu (W.D.S.)

**Keywords:** adaptive immunity, hyaluronic acid (HA), immunology, innate immunity, molecular weight (MW), wound healing

## Abstract

Hyaluronic acid (HA), a glycosaminoglycan ubiquitous in the skin, has come into the limelight in recent years for its role in facilitating dermal wound healing. Specifically, HA’s length of linearly repeating disaccharides—in other words, its molecular weight (MW)—determines its effects. High molecular weight (HMW)-HA serves an immunosuppressive and anti-inflammatory role, whereas low molecular weight (LMW)-HA contributes to immunostimulation and thus inflammation. During the inflammatory stage of tissue repair, direct and indirect interactions between HA and the innate and adaptive immune systems are of particular interest for their long-lasting impact on wound repair. This review seeks to synthesize the literature on wound healing with a focus on HA’s involvement in the immune subsystems.

## 1. Introduction

Skin injury leads to the cascade of highly interrelated processes known as wound healing, which, beginning with hemostasis, can take months to years to finish remodeling. Each year, more than 100 million people in developed countries are expected to develop scars from irregular wound healing outcomes, adding physical, financial, and psychosocial burdens to the problem [1]. Many research groups believe that the inflammation stage of wound healing plays a decisive role in modulating wound healing [2,3,4]. However, the recipe of successful wound healing is unclear due to the complexity of the immune system and the role of hyaluronan, a prolific glycosaminoglycan found in the extracellular matrix (ECM), which influences the inflammation stage of wound healing. Hyaluronic acid can exert complementary effects depending on the length of its polysaccharide chain and thus its molecular weight. Specifically, HMW-HA (>900 kDa) is anti-inflammatory [5], and LMW-HA (<120 kDa) is pro-inflammatory [6]. HA is abundant in the body, with roughly half of the total biomass of hyaluronic acid localized to unwounded skin [7,8].

This review aims to explicate the combined role of HA and various immune cell populations in dermal wound repair using relevant literature from the past decade when possible.

### Overview of HA

HA is a unique glycosaminoglycan in that it is non-sulfated and lacks a core protein, with the bulk of its structure comprising continuous residues of [glucuronate-β 1,3-N-acetylglucosamine-β 1,4-] [9]. Upon tissue injury, an upregulation of HA synthesis is driven by fibroblasts in the wound environment, while a simultaneous increase in the expression of hyaluronidases works to degrade this HA into LMW fragments [10,11]. Differences in molecular structure of HA naturally give way to differences in the receptors each variant binds, as HMW-HA tends to bind CD44, and LMW-HA preferentially binds hyaluronan-mediated motility receptor (RHAMM) and toll-like receptors (TLRs 2 and 4) [12]. Nevertheless, HA of all molecular weights does bind CD44, with reversible binding at the lowest (<10 kDa) molecular weights, and binding becoming near irreversible as the MW of HA increases [13]. Despite this irreversible binding, the binding affinity of HMW-HA to CD44 is relatively weak and is largely determined by CD44 conformational changes [14]. HA-CD44 interactions also generate differential cellular effects such as proliferation and invasion based upon whether the HA is immobilized in a matrix [15]. Activated myofibroblasts in particular, utilizing hyaluronan synthase 2 (HAS2), produce extracellular matrix bound HA with an average MW of 480 kDa [16]. 

The influence of HA on immune responses also goes beyond interactions with specific HA receptors; chemokines interacting with glycosaminoglycans in the ECM modulate the immune response at areas of tissue inflammation. For example, IL-8 binds to matrix bound heparin sulfate to generate a chemoattractive gradient for neutrophils [17,18]. IL-8 also binds to hyaluronan; however, the physiologic significance of this binding has yet to be determined [19]. Other potential binding targets of HA include HABPs (hyaluronic acid binding proteins) such as inter-alpha-trypsin inhibitor (IαI) and tenascin [20], and proteoglycans such as biglycan and versican [21]. HABPs crosslink with HA and further regulate its response to wound healing. 

Among the roles of hyaluronan, perhaps the most compelling reason to examine the interactions of HA in wound healing is its role in regenerative healing. Mid-gestational fetal wounds are known to heal without a scar [22,23]. The pericellular matrix of fetal dermal fibroblasts has been demonstrated to be rich in HA [24]. IL-10, an immunomodulatory cytokine present in high levels in fetal skin, can also achieve regenerative wound healing, which is dependent upon fibroblast synthesis of hyaluronan [25]. The rest of this review will focus on how various immune cell types work with HA to influence wound repair in the skin.

## 2. Immune System

The mammalian immune system is supported by two subsystems known as the innate immune system and adaptive immune system, which work in concert to defend the body against various pathogens. While activating a response to destroy foreign invaders, a key challenge of this system is to avoid damaging the body’s own tissues and cells in the process. In regard to hyaluronan’s role in immunity, HA can promote or inhibit TLR signaling pathways, which facilitate downstream activation of the innate immune system. In terms of the adaptive immune system, antigen activation of naïve T lymphocytes increases their CD44 expression and thus their ability to bind HA and trigger the adaptive immune system [26,27,28]. Further T cell subtypes are implicated in this process as well as potentially B cells, although the literature on the latter is sparse. The rest of this review will cover these cell populations and others in greater detail and will outline how each cell type interfaces the two systems, if applicable.

## 3. Innate Immunity

The innate immune system provides the body with a first line of defense to a pathogenic invasion by mounting a rapid and robust, though nonspecific, response. Pattern recognition receptors (PRRs) enable a nonspecific reaction by recognition of damage-associated molecular patterns (DAMPs), such as DNA or heat shock proteins, or pathogen-associated molecular patterns (PAMPs), such as lipopolysaccharides, double-stranded RNA, or bacterial DNA [29]. Of the four classes of PRRs, the most well studied is the TLR, with certain TLRs recognizing certain DAMPS and/or PAMPs. Activated TLRs can stimulate production of tumor necrosis factor alpha (TNF-α) and interleukins, which induce chemokine release and subsequent migration of inflammatory cells to the wound site [30]. This further drives maturation of dendritic cells to promote T cell maturation and polarization of T helper type 1 (Th1) [31].

As primary receptors of LMW-HA, TLR activation has been speculated to produce a dose-dependent effect on wound healing, with lower concentrations noted to stimulate regeneration [32]. Further evidence of this was found in nonhealing venous ulcers that exhibited elevated levels of TLRs 2/4, in contrast to healing wounds observed with decreased concentrations of TLRs [33]. However, one study found TLR4 in the keratinocytes of wound edges and noted that mice lacking TLR4 had prolonged epithelization [34]. For reference, TLRs 2/4/7 are diffusely expressed throughout the epidermis with lighter expression toward the stratum corneum; TLR5 is mostly found in basal epidermis keratinocytes; TLR9 is diffusely expressed with lighter expression toward the basal layer [35,36,37].

The following sections will delve deeper into specific cell populations of the innate immune system. Refer to Table 1 for a complete summary.

### 3.1. Mast Cells

HA is found in great abundance in mast cell granules [46], and mast cells play a critical role in the normal wound healing mechanism. In fact, Weller and colleagues discovered that mast cell-deficient mice exhibit significant impairment of wound closure, neutrophil recruitment, and extravasation [47]. Moreover, human mast cells with expression of CD44 were observed to facilitate attachment to HA, and cultured human mast cells adhered to surfaces covered in HA [38]. This is significant as mast cells express and secrete the anti-inflammatory cytokine IL-10 [48], which has been associated with scarless wound healing and regeneration [49]. IL-10 in turn stimulates fibroblasts to secrete HMW-HA to prevent collagen deposition and inflammatory macrophage polarization [4].

### 3.2. Macrophages

Macrophages, by way of phagocytosis, promote transition of the dermal wound from a pro-inflammatory milieu to an anti-inflammatory environment [50]. Accordingly, macrophages are able to polarize to two different subtypes: a pro-inflammatory M1 cell and an anti-inflammatory M2 cell, which are seen during early and late stages of wound healing, respectively. This M1 to M2 differentiation is associated with IL-4 and IL-10, in line with HMW-HA stimulating production of immunosuppressive M2 macrophages [51]. In fact, one recent study showed that a sulfated HA/collagen hydrogel improved diabetic wound healing in mice by hindering the TLR pathway and promoting M2 polarization of macrophages [40], supporting the crosstalk of HA between inflammatory cells and the wound ECM. Conversely, LMW-HA, acting through TLRs 2/4, induces M1 macrophage differentiation during the inflammatory stage of wound healing [39]. Further evidence suggests that macrophages are the cornerstone cells of immune regulation of the inflammatory response, as the wound healing process abruptly stops in their absence [52]. Similar behavior is not common to lymphocytes or neutrophils, as wound healing will still proceed in their absence, provided the wound environment is sterile.

### 3.3. Dendritic/Langerhans Cells

In the skin, specialized dendritic cells are known as Langerhans cells. As these CD44-rich cells migrate through the dermis, they leverage the HA of nearby keratinocytes as an adhesion substrate [53]. HA promotes maturation of dendritic cells when LMW variants act as endogenous ligands of the TLR 4 pathway during the wound inflammatory response [41]. Conversely, one study found that both hapten-induced in vivo maturation of Langerhans cells and hapten-induced migration of Langerhans cells from the epidermis were hindered when HA blocking peptide was administered topically, locally, or systemically [54].

### 3.4. Other Cell Populations

Literature on the role of other immune cell types, including neutrophils and NKT cells, specifically interacting with HA in the context of wound healing is sparse. In dermal wound repair, neutrophil involvement is generally controversial as neutrophils can release enzymes such as elastase, which can be detrimental to surrounding tissue and increase the likelihood of scarring [55]. In contrast, studies have found that mice lacking the CXCR2 receptor for neutrophil chemotaxis demonstrate poor angiogenesis and epithelialization [56]. However, none of these studies mention the role of HA in this process. Similarly, although it is known that HA binds to CD44 receptors on the surface of NKT cells [42], further downstream effects of the interaction have not been elucidated.

## 4. Adaptive Immunity

The functional cells of the adaptive immune response are lymphocytes, which can be further subdivided into T and B lymphocytes. T cells direct and regulate immune responses via secretion of characteristic profiles of cytokines and chemokines, while B cells are responsible, upon activation, for the production of antigen-specific antibodies. As we will demonstrate below, more studies have been done on HA interactions with T cells in the wound environment rather than on B cell interactions.

### 4.1. T Lymphocytes

**Figure 1:**
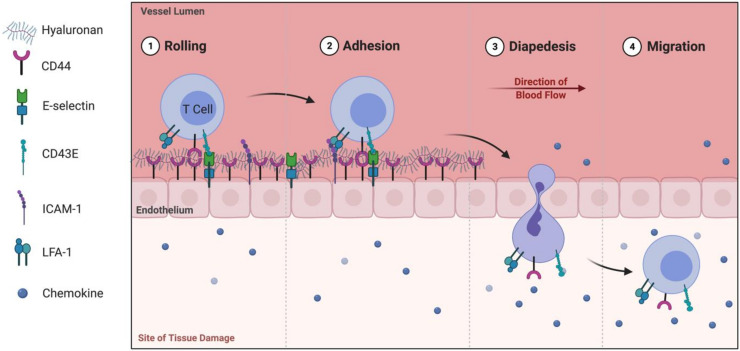
Hyaluronan/CD44 interactions involved in T lymphocyte rolling for recruitment to damaged tissue. Recruitment of leukocytes to areas of inflammation consists of four steps: (1) Rolling, (2) Adhesion, (3) Diapedesis, and (4) Migration. HA presentation on CD44 of endothelial cells serves as a substrate for T cells to interact and, in conjunction with selectin interactions, mediates rolling of the T cell, thus slowing the cell and allowing for further adhesion of LFA-1 to ICAM-1. The T cell is then able to traverse the endothelial barrier in a process called diapedesis where it migrates through tissue to participate in the inflammatory response [64,65,66]. Adapted from “Leukocyte Migration at Sites of Infection”, by BioRender.com (2021). Retrieved from app.biorender.com/biorender-templates.

Naïve T lymphocytes initially have low expression of CD44 and thus relatively little binding with HA; however, once they are activated by antigen-presenting cells and begin proliferating, their expression of CD44 expands, increasing their capacity to bind HA. [26,27]. This augmented HA binding by activated T cells likely reflects a functional change that allows T cells to infiltrate into infected or damaged tissue. T lymphocyte surface CD44 interaction with HA expressed on vascular endothelium participates in the rolling interaction of T cells, slowing the flowing cells and allowing for additional interactions between the cells and the endothelial integrins, thus facilitating adhesion and diapedesis as illustrated in Figure 1 [57,58]. Inhibition studies in an animal model of peritonitis suggests the CD44–HA interaction to be necessary for the infiltration of lymphocytes into inflamed tissue [59]. Upon invasion into inflamed tissue, lymphocytes transition from a rounded to an ameboid shape, which facilitates migration through ECM without the need for degradation by matrix metalloproteases [60]. This invasive capacity of T lymphocytes is made possible by interactions between CD44 and the ECM, facilitated by interactions between the intracellular domain of CD44 with the ezrin, radixin, and moesin (ERM) proteins that are involved in cell polarity and migration via linkage of the cellular cytoskeleton to the cell membrane [61,62,63]. T lymphocytes can be primarily divided into CD4^+^ or CD8^+^, the former involved in production of cytokines directed towards a particular immune response, and the latter responsible for destruction of virally infected host cells. Another subset of T lymphocytes is known as gamma-delta (γδ) T cells, which are able to bridge the innate and adaptive immune systems.

### 4.2. CD4^+^ T lymphocytes

Upon the arrival of activated CD4^+^ T lymphocytes, their effector or regulatory function in the wound environment is determined by the specific subtype to which they are polarized. The polarization of CD4^+^ cells is largely governed by the local inflammatory cytokine and growth factor milieu produced by innate lymphoid and tissue resident cells’ response to tissue injury or pathogen invasion [67,68,69,70]. There is some evidence for varied responses of individual lymphocyte subsets to their interactions with HA.

Regulatory T lymphocytes (Treg) express the Foxp3 transcription factor and function to curtail both innate and adaptive immune responses [71]. Treatment of Treg with HMW-HA amplified Foxp3 expression, with simultaneous increases in the production of IL-2 and immunoregulatory IL-10 and TGF-β [43]. This effect was dependent upon CD44 expression and implicates a role for CD44 crosslinking for Treg to produce regulatory cytokines, as neither LMW-HA nor soluble anti-CD44 antibodies were able to recapitulate this outcome. The capacity of Treg to produce IL-10, TGF-β, and IFNγ also requires expression of active CD44, which is capable of binding HA, as opposed to Treg, which express inactive CD44 [72]. These studies establish a role for the production of HMW-HA in wounds as a factor, which promotes the immunosuppressive capacity of wound-infiltrating Treg to cull the inflammatory response and encourage a reparative environment.

Less is known of the interaction between type 1 or 2 helper T cells (Th1/Th2) and hyaluronan in the wound environment. In vitro differentiated Th1 and Th2 demonstrate a somewhat higher binding capacity to HA when compared to naïve T lymphocytes [73]. Despite only a marginal increase in binding capability, Th1 and Th2 are still able to undergo HA-mediated rolling interactions with endothelium, thus allowing for recruitment to inflamed or damaged tissues [73].

### 4.3. Gamma Delta T (γδ T) Cells

γδ T cells are a group of T lymphocytes that demonstrate behavior that blurs the line between being the first line of defense in innate immunity and coordinating a targeted response along with B cells and more conventional T cells as part of adaptive immunity. Regarding innate immunity, studies have shown phagocytic capabilities of human Vγ9/Vδ2 T cells, a feature previously thought to be solely the domain of innate myeloid lineage cells [74]. In the adaptive immune system, γδ T cells can rearrange γ and δ genes through a hallmark process known as V(D)J recombination to develop a memory phenotype for future recognition [75]. In mice, a subpopulation of γδ T cells known as dendritic epidermal T cells, or DETCs, can retract their dendrites in response to keratinocyte damage. Growth factors like keratinocyte growth factors 1 and 2 are then secreted, which stimulate HA production and subsequent recruitment of macrophages to the wound site [44].

### 4.4. B Cells

The role of B lymphocytes in wound healing is not well studied, and thus the literature regarding HA–B cell interactions in wound healing is lacking. In a mouse model of B cell deficient mice, wound healing was delayed with a decreased inflammatory cell infiltration [45]. Similarly, the topical application of B cells to wounds of diabetic mice resulted in accelerated closure [76]. HA application to murine wounds also serves to attract B cells to the wound bed, leading to activation via TLR4 and the production of IL-6 and TGF-β, though the molecular size of this HA was not specified [45]. Further work is still needed to determine the specific molecular weight-based interactions between HA and B cells in the wound environment.

## 5. Concluding Thoughts

The human body’s defense system against outside intruders is a marvel of well-orchestrated mechanisms. In dermal wound healing, the interactions of HA with the innate and adaptive immune systems help regulate the skin’s response to injury, fine-tuned by the high and low molecular weight variants of HA. In the innate immune system, the interaction of LMW-HA with decreased levels of TLRs 2/4 were associated with healing wounds, although a lack of TLR 4 altogether resulted in delayed epithelialization. Moreover, innate immune cells such as macrophages, mast cells, and dendritic/Langerhans cells further regulate wound repair by leveraging HA-dependent mechanisms. In the adaptive immune system, B cells generate antigen-specific antibodies upon activation, and T cells mediate immune responses by their secretion of cytokines and chemokines. For example, augmentation of Foxp3 expression of Tregs by HMW-HA leads to the production of anti-inflammatory cytokines such as IL-2, IL-10, and growth factors such as TGF-β. T cell subsets, such as γδ T cells, toe the line of conventional immune cells and exhibit properties of both subsystems depending on their mechanism of action. Similarly, the mammalian complement system works alongside dynamic cell types such as γδ T cells and NKT cells to enhance the immune response. In sum, this review serves as a reference and tool for researchers to understand the current literature on the role of the immune system and extracellular matrix biology in the context of wound healing in the skin.

## Figures and Tables

**Table 1:** Summary of the various immune cell types and associated subtypes and their role in wound healing in the context of HA.

Immune Subsystem	Cell Type	Subtype	Role with HA in Wound Healing
Innate	Mast cells		Facilitate attachment to HA [38] and promote anti-inflammatory wound milieu in concert with HA by way of IL-10 [4]
	Macrophages	M1	Induced by LMW-HA during inflammatory stage of wound healing [39]
		M2	Sulfated HA/collagen hydrogel improved murine diabetic wound healing by promoting M2 macrophages [40]
	Dendritic/Langerhans cells		LMW-HA promotes maturation of dendritic cells via TLR 4 pathway during inflammatory phase of wound healing [41]
	Natural killer T (NKT) cells		HA binds to CD44 receptors on the surface of NKT cells although downstream effects are unknown [42]
Adaptive	T lymphocytes		Antigen activation of T lymphocytes induces HA binding via CD44 which enables lymphocyte infiltration into inflamed tissue [28]
		CD4^+^	HMW-HA amplifies Foxp3 expression of Tregs which stimulates production of IL-2 and immunosuppressive IL-10 and TGF-β [43]
		γδ	Subpopulation known as DETCs secrete keratinocyte growth factors 1 and 2 which stimulate HA production and macrophage recruitment [44]
	B cells		HA application to murine wounds attracts B cells to wound bed, stimulating production of IL-6 and TGF-β [45]

## Glossary

DAMP	damage-associated molecular pattern
DETC	dendritic epidermal T cells
ECM	extracellular matrix
γδ T	gamma delta T cell
HA	hyaluronic acid/hyaluronan
HMW-HA	high molecular-weight hyaluronic acid
IL	interleukin
LMW-HA	low molecular-weight hyaluronic acid
MW	molecular weight
NKT	natural killer T
PAMP	pathogen-associated molecular pattern
PRR	pattern recognition receptor
RHAMM	receptor for HA-mediated motility
TCR	T cell receptor
TGF-β	transforming growth factor beta
Th1/2	T helper type 1/2
TLR	toll-like receptor
TNF-α	tumor necrosis factor alpha
